# 

**Published:** 1861-02

**Authors:** 


### RECEIPTS NOT HERETOFORE ACKNOWLEDGED.

| | |
|---|---|
| Dr. P A Rosenberger, Ill., vol. 4 | $4 00 |
| " Edward L H Barry, Ill., vol. 4 | 1 00 |
| " Alex. Hiltsie, Iowa, vol. 4 | 2 00 |
| " I N Allen, Ill., vol. 4 | 2 00 |
| " S C Gray, Ind., vol. 3 | 2 00 |
| " J B Felker, Ill., vol. 4 | 2 00 |
| " C S Tucker, Mich , vol. 4 | 2 00 |
| " D Kirkpatrick, Ind., vol. 4 | 1 00 |
| " Kittle, Iowa, vol. 4 | 2 00 |
| Dr. J R Webster, Ill., vol. 4 | 2 00 |
| " Wm. Law, Ill., vol. 3 | 2 00 |
| " J T Pearman, Ill., vol. 4 | 2 00 |
| " C Goodbrake, Ill., vol. 4 | 2 00 |
| " L A Engle, Ill., vol. 4 | 2 00 |
| " B Wilson, Ill., vol. 4 | 2 00 |
| " J M Bower, Ill., vol 4 | 2 00 |
| " —— Holmes, Ill., vol. 4 | 2 00 |

